# Electroencephalography characteristics related to risk of sudden unexpected death in epilepsy in patients with Dravet syndrome

**DOI:** 10.3389/fneur.2023.1222721

**Published:** 2023-09-07

**Authors:** Jeong-Youn Kim, Jeongyoon Shin, Laehyun Kim, Se Hee Kim

**Affiliations:** ^1^Electronics and Telecommunication Research Institute (ETRI), Daejeon, Republic of Korea; ^2^School of Electrical and Electronic Engineering, College of Engineering, Yonsei University, Seoul, Republic of Korea; ^3^Yonsei Biomedical Research Institute, College of Medicine, Yonsei University, Seoul, Republic of Korea; ^4^Center for Bionics, Korea Institute of Science and Technology, Seoul, Republic of Korea; ^5^Department of HY-KIST Bio-Convergence, Hanyang University, Seoul, Republic of Korea; ^6^Pediatric Neurology, Department of Pediatrics, Epilepsy Research Institute, Severance Children’s Hospital, Yonsei University College of Medicine, Seoul, Republic of Korea

**Keywords:** epilepsy, Dravet syndrome (DS), sudden unexpected death in epilepsy (SUDEP), electroencephalography (EEG), mortality

## Abstract

**Objective:**

To investigate the quantitative electroencephalography (EEG) features associated with a high risk of sudden unexpected death in epilepsy (SUDEP) in patients with Dravet syndrome (DS).

**Methods:**

Patients with DS and healthy controls (HCs) who underwent EEG were included in the study. EEG signals were recorded using a 21 channel digital EEG system, and pre-processed data were analyzed to identify quantitative EEG features associated with a high SUDEP risk. To assess the risk of SUDEP, SUDEP-7 scores were used.

**Results:**

A total of 64 patients with DS [38 males and 26 females, aged: 128.51 ± 75.50 months (range: 23–380 months)], and 13 HCs [7 males and 6 females, aged: 95.46 ± 86.48 months (range: 13–263 months)] were included. For the absolute band power, the theta power was significantly higher in the high-SUDEP group than in the low-SUDEP group in the central brain region. For the relative band power, the theta power was also significantly higher in the high-SUDEP group than in the low-SUDEP group in the central and occipital brain regions. The alpha power was significantly lower in the high-SUDEP group than in the low-SUDEP group in the central and parietal brain regions.

**Conclusion:**

Patients with high SUDEP-7 scores have different EEG features from those with low SUDEP-7 scores, suggesting that EEG may be used as a biomarker of SUDEP in DS.

**Significance:**

Early intervention in patients with DS at a high risk of SUDEP can reduce mortality and morbidity. Patients with high theta band powers warrant high-level supervision.

## 1. Introduction

Dravet syndrome (DS) is an SCN1A mutation-related, infantile-onset epilepsy syndrome, characterized by a distinctive seizure history: prolonged febrile or afebrile seizures beginning in the first year of life, followed by subsequent multiple seizure types. Patients with DS experience developmental regression during early childhood, mostly because of frequent pharmaco-resistant seizures (1–3). Early diagnosis of DS is critical to avoid anticonvulsants that may aggravate seizures and increase morbidity (4).

Premature mortality is a leading cause of fear among caregivers (5–7). Up to 15% of patients with DS die during early childhood or adolescence (1), and most of them experience SUDEP (1), which is defined as sudden, unexpected, witnessed or unwitnessed, non-traumatic, and non-drowning death in patients with epilepsy (8). SUDEP accounts for 7.5%–17% of all deaths of epilepsy (9, 10), but SUDEP rates are particularly high in patients with DS attributing to 20% of all deaths (11). The mean age of SUDEP is as young as 4.6 years in patients with DS (6), emphasizing the high risk of mortality.

SUDEP is a diagnosis of exclusion (12), as the definitive post-mortem signs or biomarkers of SUDEP have not yet been identified (13, 14). Indirect evidence has linked SUDEP to seizure-induced apnea, pulmonary edema, dysregulation of cerebral circulation, and cardiac arrhythmias (9, 10, 15), which may occur secondary to hormonal or metabolic changes or autonomic discharges (9, 15, 16), but the exact mechanisms remain unclear.

Electroencephalography (EEG) is a critical tool that can show the clinical status of patients, as well as ictal changes (17, 18) and age-related changes in patients with DS (19). Recently, few studies have analyzed the risk of SUDEP using EEG in patients with various epilepsies. One recent multi-center study has suggested that machine learning-driven models may be used to quantify SUDEP risk in patients with epilepsy (20). Another study used the data of a group of patients with drug-resistant epilepsy to compare the EEG and ECG data of 21 patients with definite or probable SUDEP, and it reported an increased autonomic stimulation associated with seizures in patients with SUDEP (21). However, these studies included patients with various epilepsies, and the results were largely dependent on the ECG data (20, 21).

In the present study, we performed a quantitative EEG analysis to identify the EEG features related to a high risk of SUDEP. We hypothesized that the EEG features would be different between patients with high SUDEP scores and the others with lower SUDEP scores.

## 2. Methods

### 2.1. Participants

This study was performed at Severance Children’s Hospital. We enrolled patients who were diagnosed with DS between 1 January, 2012 and 31 March, 2021. DS was diagnosed according to the following criteria: (1) febrile or afebrile, generalized or unilateral, or clonic or tonic-clonic seizures that occur in the first year of life in an otherwise healthy infant; (2) the development of drug-resistant myoclonus, atypical absences, and focal seizures; (3) a developmental delay within the second year of life; (4) the subsequent development of cognitive impairment or other neuropsychiatric and behavioral disorders; and (5) an identified SCN1A gene mutation. Only patients who recently underwent EEG after 1 January, 2020 were included. EEGs were performed as routine clinical care to evaluate seizures and the background EEG activity. The healthy controls (HCs) group included patients who came to the neurologic outpatient clinic due to non-epileptic events such as dizziness or headache. Based on the SUDEP-7 inventory score, patients with DS were then divided into low- (score: 0–3), mid- (score: 4–6), and high-SUDEP (score 7–9) subgroups. This study was approved by the institutional review board (4-2021-0377). Informed consent was waived because of the retrospective nature of the study, and we used anonymous clinical data.

### 2.2. SUDEP-7 score

The SUDEP-7 inventory was assembled from the large prospective cohort study of SUDEP reported by Walczak et al. (22). The core risk factors identified by Walczak et al. (22) were consolidated into a seven-item inventory. Risk factors with low odds ratios (0–2) were not included. The risk factor “any seizures, average per month” was consolidated into two core risk factors: any seizures in the last year or more than 50 seizures per month. The weighting for each risk factor was determined by the natural log of the odds ratio rounded to the nearest integer. The weighted SUDEP-7 inventory was scored from 0 to 12. The seven items were shown in Table 1.

**Table 1:** SUDEP-7 score inventory.

	SUDEP risk factors
1	More than 3 tonic-clonic seizures in last year
2	1 or more tonic-clonic seizures in last year
3	One or more seizures of any type over the last 12 months
4	>50 seizures of any type per month over the last 12 months
5	Duration of epilepsy ≥30 years
6	Current use of three or more anti-epileptic drugs
7	Mental retardation, I.Q. <70, or too impaired to test

SUDEP, sudden unexpected death in epilepsy.

### 2.3. EEG acquisition and analysis

The patients lay on an examination bed in a room with ambient noise blocked. EEGs were recorded for at least 30 min. The EEG signals were recorded using a 21-channel digital EEG system (Xltek, Natus Medical Incorporated, San Carlos, California or Telefactor Aurora^®^ EEG machine, Grass-Telefactor, Melbourne, Australia). The electrodes were attached according to the international 10–20 system. Data were recorded using a sampling rate of 200 or higher with filter settings of 1–70 Hz. Epochs with too many artefacts were removed from the recorded data by visual inspection. The pre-processed EEG data was divided into multiple epochs of a length of 2 s. Power spectral analysis was used to compress the rhythmic information of the brain wave signals. In the power spectral analysis, the periodogram function in MATLAB R2020a (MathWorks, Natick, MA, United States) was used to calculate the power spectral density of each epoch. The spectral absolute and relative powers were then averaged according to randomly selected 30 epochs.

The absolute band powers were classified into five frequency bands: delta (1–4 Hz), theta (4–8 Hz), alpha (8–12 Hz), beta (12–30 Hz), and gamma (30–50 Hz). The relative band powers were calculated by dividing the absolute band powers by the total power of 1–50 Hz. The powers were averaged into six regions: frontal (FP1, FP2, F7, F8, F3, F4, and Fz), central (C3, C4, and Cz), temporal (T3, T4, T5, and T6), parietal (P3, P4, and Pz), and occipital (O1 and O2).

### 2.4. Statistical analysis

Independent *t*-tests were used to compare the demographic data. A multivariate analysis of covariance (MANCOVA) was used to compare the absolute and relative EEG band powers between patients with DS and HCs. Age was controlled for as a covariate.

The MANCOVA was used to compare the absolute and relative EEG band powers among the SUDEP subgroups (low-, mid-, and high-SUDEP) of patients with DS. *p*-values were adjusted for age. Statistical analyses were performed using SPSS 21 (SPSS, Inc., Chicago, IL, United States).

## 3. Results

A total 64 patients with DS [38 males and 26 females, aged: 128.51 ± 75.50 months (range: 23–380 months)] were included in this study. A total of 13 HCs [7 males and 6 females, aged: 95.46 ± 86.48 months (range: 13–263 months)] were included in this study.

Low-SUDEP group consisted of 14 patients [10 males and 4 females, aged: 122.73 ± 54.10 months (range: 68–238 months)], mid-SUDEP, 14 [6 males and 8 females, aged: 113.83 ± 76.73 months (range: 55–380 months)] and high-SUDEP, 31 [19 males and 12 females, aged: 138.51 ± 81.81 months (range: 23–347 months)]. Five patients were excluded because of lacking information on clinical data.

There was a difference in age between patients with DS and HCs (*p* = 0.021), and other demographics are no different.

Demographic data of the DS patients and the HCs are reported in Table 2.

**Table 2:** Demographic data of Dravet syndrome (DS) patients, healthy controls (HCs), and SUDEP subgroups.

Group	*N*	Male	Female	Age (months) Mean (*SD*)
DS	64	38	26	128.51 (75.50)
HCs	13	7	6	95.46 (86.48)
Low-SUDEP	14	10	4	122.73 (54.10)
Mid-SUDEP	14	6	8	113.83 (76.73)
High-SUDEP	31	19	12	138.51 (81.81)

### 3.1. Differences in EEG between the DS and HCs groups

The multivariate analysis of covariance (MANCOVA) was applied to the absolute and relative EEG power between patients with DS and HCs. Age was controlled as a covariate.

For the absolute band power, the delta power was significantly lower in the DS group than in the HCs group in the occipital region [68.19 (31.81; 150.75) vs. 124.68 (34.98; 280.48), *p* = 0.033]. The beta power was also significantly higher in the DS group than in the HCs group in the following sub-regions: frontal [17.96 (12.69; 34.32) vs. 11.01 (7.82; 12.27), *p* = 0.008], temporal [18.24 (12.89; 31.14) vs. 12.48 (10.03; 14.08), *p* = 0.010], central [16.14 (9.36; 25.35) vs. 8.52 (5.88; 10.94), *p* = 0.003], and parietal [13.96 (10.00; 22.69) vs. 9.45 (7.57; 11.59), *p* = 0.010]. All the comparison results of the absolute EEG band power between the DS and HCs groups are presented in Table 3.

**Table 3:** Comparison of the absolute electroencephalography (EEG), power between patients with Dravet syndrome (DS) and healthy controls (HCs).

Frequency band	Brain region	DS (*n* = 64)	HCs (*n* = 13)	*p*
		Median [*Q*1; *Q*3]		
Delta	Frontal	55.44 [33.47; 117.92]	60.16 [33.68; 159.96]	0.689
	Temporal	49.46 [25.07; 97.38]	63.34 [27.37; 149.68]	0.084
	Central	42.96 [17.90; 68.65]	49.39 [13.04; 143.81]	0.238
	Parietal	37.16 [18.72; 82.39]	65.83 [15.47; 122.14]	0.084
	Occipital	68.19 [31.81; 150.75]	124.68 [34.98; 280.48]	0.033^*^
Theta	Frontal	37.44 [19.91; 59.38]	41.34 [24.75; 58.08]	0.233
	Temporal	43.79 [18.54; 69.81]	45.90 [26.48; 96.63]	0.860
	Central	36.92 [18.85; 61.45]	34.50 [22.35; 59.60]	0.145
	Parietal	43.63 [17.40; 71.68]	43.04 [29.20; 93.06]	0.889
	Occipital	51.73 [29.44; 100.21]	111.94 [58.29; 159.39]	0.577
Alpha	Frontal	13.17 [8.25; 21.67]	14.10 [13.46; 25.21]	0.555
	Temporal	15.94 [8.38; 29.56]	27.31 [13.22; 35.83]	0.996
	Central	11.79 [7.75; 19.49]	17.75 [10.50; 25.76]	0.257
	Parietal	12.39 [7.87; 23.35]	22.19 [12.36; 39.50]	0.337
	Occipital	24.64 [12.44; 53.54]	72.08 [26.11; 152.33]	0.089
Beta	Frontal	17.96 [12.69; 34.32]	11.01 [7.82; 12.27]	0.008^*^
	Temporal	18.24 [12.89; 31.14]	12.48 [10.03; 14.08]	0.010^*^
	Central	16.14 [9.36; 25.35]	8.52 [5.88; 10.94]	0.003^*^
	Parietal	13.96 [10.00; 22.69]	9.45 [7.57; 11.59]	0.010^*^
	Occipital	19.89 [13.66; 37.68]	20.79 [17.01; 25.82]	0.238
Gamma	Frontal	2.67 [1.99; 4.00]	4.00 [2.30; 5.12]	0.829
	Temporal	2.80 [1.89; 4.20]	2.65 [2.58; 4.13]	0.455
	Central	1.79 [1.20; 2.78]	1.80 [1.42; 2.10]	0.059
	Parietal	1.85 [1.16; 2.77]	1.88 [1.21; 2.26]	0.130
	Occipital	2.89 [1.80; 4.08]	3.24 [2.41; 4.32]	0.541

^*^p < 0.05. EEG, electroencephalography.

For the relative band power, the theta power in the occipital region was significantly higher in the DS group than in the HCs group [0.27 (0.23; 0.33) vs. 0.22 (0.16; 0.27), *p* = 0.013]. The alpha power was also significantly lower in the DS group than in the HCs group in the following sub-regions: temporal [0.11 (0.08; 0.16) vs. 0.21 (0.05; 0.24), *p* = 0.045], central [0.11 (0.08; 0.15) vs. 0.20 (0.05; 0.29), *p* = 0.001], parietal [0.12 (0.07; 0.17) vs. 0.24 (0.05; 0.30), *p* = 0.001], and occipital [0.13 (0.09; 0.21) vs. 0.30 (0.05; 0.43), *p* = 0.006]. The gamma band power in the frontal region was significantly lower in the DS group than in the HCs group [0.02 (0.01; 0.03) vs. 0.03 (0.01; 0.04), *p* = 0.010]. All the comparison results of the relative EEG band power between the DS and HCs groups are presented in Table 4. No other frequency bands in the brain regions showed any significant differences between the DS and HCs groups.

**Table 4:** Comparison of the relative EEG power between patients with Dravet syndrome (DS) and healthy controls (HCs).

Frequency band	Brain region	DS (*n* = 64)	HCs (*n* = 13)	*p*
		Median [*Q*1; *Q*3]		
Delta	Frontal	0.41 [0.31; 0.52]	0.43 [0.39; 0.56]	0.481
	Temporal	0.38 [0.30; 0.46]	0.36 [0.31; 0.69]	0.808
	Central	0.34 [0.25; 0.43]	0.31 [0.28; 0.60]	0.936
	Parietal	0.37 [0.25; 0.43]	0.31 [0.27; 0.52]	0.771
	Occipital	0.41 [0.26; 0.47]	0.26 [0.21; 0.74]	0.315
Theta	Frontal	0.24 [0.19; 0.32]	0.22 [0.18; 0.30]	0.358
	Temporal	0.28 [0.22; 0.35]	0.25 [0.19; 0.29]	0.201
	Central	0.30 [0.23; 0.39]	0.26 [0.19; 0.32]	0.094
	Parietal	0.29 [0.24; 0.40]	0.26 [0.19; 0.33]	0.127
	Occipital	0.27 [0.23; 0.33]	0.22 [0.16; 0.27]	0.013^*^
Alpha	Frontal	0.09 [0.07; 0.13]	0.13 [0.06; 0.17]	0.084
	Temporal	0.11 [0.08; 0.16]	0.21 [0.05; 0.24]	0.045^*^
	Central	0.11 [0.08; 0.15]	0.20 [0.05; 0.29]	0.001^*^
	Parietal	0.12 [0.07; 0.17]	0.24 [0.05; 0.30]	0.001^*^
	Occipital	0.13 [0.09; 0.21]	0.30 [0.05; 0.43]	0.006^*^
Beta	Frontal	0.14 [0.09; 0.27]	0.08 [0.03; 0.12]	0.119
	Temporal	0.12 [0.09; 0.26]	0.08 [0.03; 0.12]	0.291
	Central	0.14 [0.10; 0.27]	0.06 [0.03; 0.12]	0.252
	Parietal	0.12 [0.07; 0.23]	0.07 [0.03; 0.11]	0.291
	Occipital	0.10 [0.07; 0.20]	0.05 [0.02; 0.09]	0.950
Gamma	Frontal	0.02 [0.01; 0.03]	0.03 [0.01; 0.04]	0.010^*^
	Temporal	0.02 [0.01; 0.03]	0.02 [0.01; 0.04]	0.399
	Central	0.02 [0.01; 0.03]	0.02 [0.01; 0.02]	0.480
	Parietal	0.01 [0.01; 0.02]	0.01 [0.01; 0.02]	0.730
	Occipital	0.01 [0.01; 0.02]	0.01 [0.01; 0.01]	0.379

^*^p < 0.05. EEG, electroencephalography.

### 3.2. Differences in EEG between the low-SUDEP and high-SUDEP group

The MANCOVA was applied to the absolute and relative EEG power between the low-SUDEP and high-SUDEP groups. Age was controlled for as a covariate.

For the absolute band power, the theta power was significantly higher in the high-SUDEP group than in the low-SUDEP group in the central brain regions [23.60 (11.30; 44.02) vs. 47.16 (19.37; 61.79), *p* = 0.042]. All the comparison results of the absolute EEG band power between the high-SUDEP and low-SUDEP groups are presented in Table 5.

**Table 5:** Comparison of the absolute EEG power among low-SUDEP, mid-SUDEP, and high-SUDEP groups in patients with Dravet syndrome.

Frequency band	Brain region	Low-SUDEP (*n* = 14)	Mid-SUDEP (*n* = 14)	High-SUDEP (*n* = 31)	*p*
		Median [*Q*1; *Q*3]			
Delta	Frontal	54.47 [30.82; 79.93]	54.26 [41.80; 107.24]	55.56 [26.06; 124.63]	0.429
	Temporal	40.70 [19.14; 74.55]	51.92 [33.82; 89.09]	51.40 [24.14; 105.55]	0.349
	Central	30.31 [13.49; 47.97]	43.82 [25.13; 66.83]	40.78 [14.82; 82.93]	0.275
	Parietal	34.06 [16.35; 48.55]	43.26 [25.02; 66.52]	41.62 [17.07; 105.55]	0.254
	Occipital	80.19 [23.62; 143.77]	92.17 [52.60; 126.66]	51.51 [30.44; 150.82]	0.315
Theta	Frontal	26.30 [12.91; 46.23]	35.79 [22.11; 56.74]	42.42 [26.29; 68.05]	0.177
	Temporal	29.93 [13.34; 72.51]	48.37 [19.73; 73.58]	47.52 [23.96; 69.71]	0.873
	Central	23.60 [11.30; 44.02]	39.03 [25.17; 67.57]	47.16 [19.37; 61.79]	0.042^*^
	Parietal	22.40 [11.41; 64.88]	47.25 [22.80; 77.95]	52.04 [18.00; 71.24]	0.531
	Occipital	42.94 [27.12; 87.56]	56.63 [34.67; 133.01]	54.35 [28.65; 93.89]	0.950
Alpha	Frontal	12.68 [8.39; 23.32]	12.16 [8.04; 19.59]	13.45 [8.55; 23.77]	0.644
	Temporal	20.11 [11.55; 32.30]	17.32 [8.13; 34.32]	13.59 [8.03; 28.81]	0.404
	Central	11.97 [9.10; 19.57]	10.93 [7.18; 20.68]	11.66 [8.51; 21.14]	0.986
	Parietal	15.98 [11.00; 20.77]	12.51 [8.25; 30.34]	11.71 [7.39; 25.49]	0.531
	Occipital	37.66 [24.55; 70.32]	24.11 [13.37; 67.73]	22.63 [12.67; 38.64]	0.288
Beta	Frontal	14.07 [11.99; 33.70]	21.06 [10.65; 25.01]	22.06 [13.79; 43]	0.056
	Temporal	20.77 [12.28; 29.89]	20.11 [15.02; 29.87]	17.40 [12.94; 29.16]	0.070
	Central	11.73 [8.32; 18.46]	15.17 [10.05; 29.73]	16.58 [9.83; 25.81]	0.079
	Parietal	14.45 [8.25; 21.65]	13.19 [11.28; 27.19]	14.35 [9.69; 21.43]	0.101
	Occipital	23.35 [14.36; 39.21]	25.3 [15.61; 42.15]	18.09 [10.91; 37.71]	0.242
Gamma	Frontal	2.51 [2.27; 3.06]	2.69 [1.98; 3.17]	3.07 [1.95; 4.29]	0.608
	Temporal	2.84 [1.97; 3.92]	3.77 [2.48; 4.57]	2.71 [1.49; 5.02]	0.413
	Central	1.92 [1.29; 2.10]	2.38 [1.49; 2.89]	1.44 [1.06; 3.19]	0.209
	Parietal	1.65 [1.20; 2.15]	2.03 [1.59; 2.73]	1.87 [1.09; 3.09]	0.368
	Occipital	2.86 [2.33; 3.64]	3.35 [2.82; 4.58]	2.57 [1.24; 4.18]	0.234

^*^p < 0.05. EEG, electroencephalography; SUDEP, sudden unexpected death in epilepsy.

For the relative band power, the theta power was significantly higher in the high-SUDEP group than in the low-SUDEP group in the following sub-regions: central [0.24 (0.19; 0.39) vs. 0.31 (0.26; 0.39), *p* = 0.044] and occipital [0.24 (0.17; 0.29) vs. 0.30 (0.23; 0.35), *p* = 0.021]. The alpha power was also significantly lower in the high-SUDEP group than in the low-SUDEP group in the following sub-regions: central [0.13 (0.12; 0.17) vs. 0.10 (0.07; 0.15), *p* = 0.032] and parietal [0.15 (0.13; 0.20) vs. 0.10 (0.07; 0.15), *p* = 0.023]. All the comparison results of the relative EEG band power between the high-SUDEP and low-SUDEP groups are presented in Table 6. No other frequency bands or brain regions showed any significant differences between the low-SUDEP and high-SUDEP groups.

**Table 6:** Comparison of the relative EEG power among low-SUDEP, mid-SUDEP, and high-SUDEP groups in patients with Dravet syndrome.

Frequency band	Brain region	Low-SUDEP (*n* = 14)	Mid-SUDEP (*n* = 14)	High-SUDEP (*n* = 31)	*p*
		Median [*Q*1; *Q*3]			
Delta	Frontal	0.44 [0.27; 0.51]	0.45 [0.34; 0.53]	0.39 [0.30; 0.46]	0.320
	Temporal	0.34 [0.28; 0.44]	0.42 [0.31; 0.49]	0.37 [0.29; 0.45]	0.272
	Central	0.33 [0.26; 0.39]	0.37 [0.26; 0.46]	0.34 [0.24; 0.41]	0.429
	Parietal	0.33 [0.26; 0.40]	0.42 [0.26; 0.43]	0.36 [0.25; 0.42]	0.453
	Occipital	0.37 [0.22; 0.45]	0.41 [0.28; 0.48]	0.37 [0.26; 0.48]	0.773
Theta	Frontal	0.20 [0.15; 0.27]	0.26 [0.23; 0.32]	0.24 [0.20; 0.34]	0.138
	Temporal	0.24 [0.17; 0.32]	0.28 [0.23; 0.34]	0.29 [0.24; 0.36]	0.128
	Central	0.24 [0.19; 0.39]	0.32 [0.27; 0.41]	0.31 [0.26; 0.39]	0.044^*^
	Parietal	0.24 [0.18; 0.40]	0.33 [0.27; 0.43]	0.30 [0.28; 0.40]	0.066
	Occipital	0.24 [0.17; 0.29]	0.27 [0.24; 0.30]	0.30 [0.23; 0.35]	0.021^*^
Alpha	Frontal	0.10 [0.09; 0.15]	0.09 [0.07; 0.14]	0.08 [0.06; 0.12]	0.249
	Temporal	0.14 [0.11; 0.23]	0.11 [0.08; 0.16]	0.10 [0.07; 0.15]	0.059
	Central	0.13 [0.12; 0.17]	0.10 [0.08; 0.14]	0.10 [0.07; 0.15]	0.032^*^
	Parietal	0.15 [0.13; 0.20]	0.12 [0.09; 0.16]	0.10 [0.07; 0.15]	0.023^*^
	Occipital	0.20 [0.15; 0.26]	0.14 [0.11; 0.19]	0.13 [0.08; 0.19]	0.127
Beta	Frontal	0.14 [0.07; 0.39]	0.13 [0.10; 0.20]	0.14 [0.10; 0.28]	0.410
	Temporal	0.14 [0.07; 0.35]	0.13 [0.11; 0.18]	0.12 [0.09; 0.24]	0.569
	Central	0.13 [0.07; 0.32]	0.16 [0.12; 0.19]	0.14 [0.10; 0.27]	0.567
	Parietal	0.13 [0.06; 0.32]	0.14 [0.10; 0.20]	0.11 [0.08; 0.24]	0.545
	Occipital	0.09 [0.05; 0.25]	0.11 [0.08; 0.15]	0.11 [0.08; 0.19]	0.913
Gamma	Frontal	0.02 [0.01; 0.03]	0.02 [0.01; 0.03]	0.02 [0.01; 0.03]	0.347
	Temporal	0.02 [0.02; 0.03]	0.02 [0.02; 0.03]	0.02 [0.01; 0.04]	0.841
	Central	0.03 [0.01; 0.04]	0.02 [0.01; 0.02]	0.01 [0.01; 0.02]	0.236
	Parietal	0.02 [0.01; 0.03]	0.01 [0.01; 0.02]	0.01 [0.01; 0.02]	0.543
	Occipital	0.01 [0.01; 0.02]	0.02 [0.01; 0.02]	0.01 [0.01; 0.02]	0.532

^*^p < 0.05. EEG, electroencephalography; SUDEP, sudden unexpected death in epilepsy.

**Figure 1:**
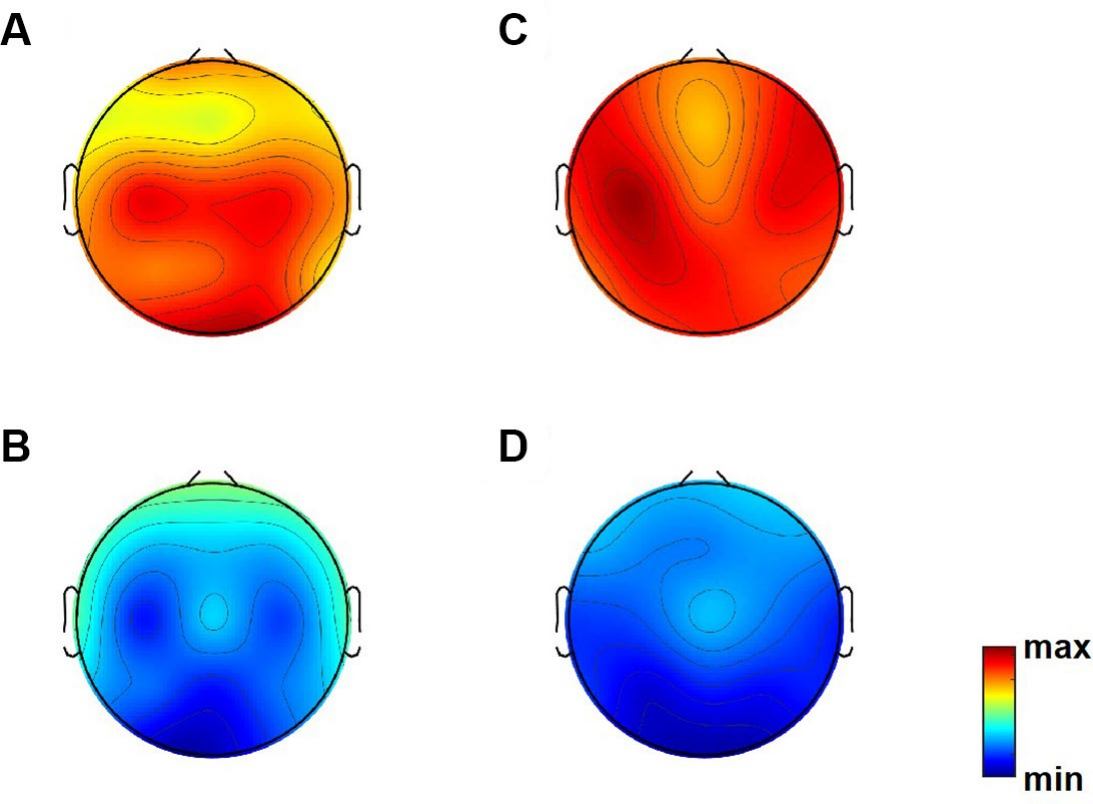
Topographic maps. **(A)** Differences of relative power in theta band between Dravet syndrome (DS) and healthy controls (HCs) groups (DS−HCs); **(B)** differences of relative power in alpha band (DS−HCs); **(C)** differences of relative power in theta band between high-SUDEP and low-SUDEP groups (high-SUDEP−low-SUDEP); **(D)** differences of relative power in alpha band (high-SUDEP−low-SUDEP).

Figure 1 presents topographic maps. (A) Differences of relative power in theta band between Dravet syndrome (DS) and healthy controls (HCs) groups (DS−HCs); (B) differences of relative power in alpha band (DS−HCs); (C) differences of relative power in theta band between high-SUDEP and low-SUDEP groups (high-SUDEP−Low-SUDEP); (D) differences of relative power in alpha band (high-SUDEP−low-SUDEP).

## 4. Discussion

SUDEP is a fatal condition that can occur frequently in patients with DS. However, biomarker for predicting SUDEP remains to be investigated. This study demonstrates that patients with high SUDEP-7 scores have different EEG features from those with low SUDEP-7 scores, suggesting that EEG may be used as a biomarker of SUDEP in DS.

The most significant finding of this study was the increase in the theta powers in patients with high SUDEP-7 scores. At the same time, the relative alpha powers of EEG signals decreased in the same group. In previous studies, the 1–30 Hz-frequency range and theta activity (4–7 Hz) showed a consistent relationship with epilepsy. Topographically a diffuse increase in theta activity characterizes the broad spectrum of genetic-developmental, non-lesional childhood epilepsies (23, 24), idiopathic generalized epilepsies (25), and focal epilepsies with dissimilar aetiology (26, 27) compared to the theta activity in healthy controls. Previous studies have reported a diffuse theta, and delta slowing in DS (17, 28). Modifications of theta and delta rhythms have been reported in SCN1a knock-out mice (29).

Notably, the alpha band powers decreased in DS patients with high SUDEP scores. Alpha-band oscillations play an important role in information processing. Alpha band rhythm increases attention by inhibiting task-irrelevant processes (30–33). In epilepsy, a slower alpha rhythm is associated with poorer seizure control (34). These findings suggest that the dysregulation of alpha activities, which represent cognitive activities, is related to a high SUDEP risk score. Intellectual disability has been suggested as a factor associated with a high SUDEP risk, but data about this hypothesis is still limited (35). Our findings correlate with these previous findings, and demonstrate that alpha is decreased in high-SUDEP group using a quantitative EEG analysis. Our findings suggest that an increased theta and alpha activity may be used to predict an increased risk of SUDEP.

In this study, we also compared the EEG findings of patients with DS to those of HCs. This study showed that absolute beta activities increased diffusely in patients with DS, in which occipital delta activities decreased in patients with DS. The relative alpha and gamma powers of EEG signals decreased in the same group. An increase in diffuse beta activity might be associated with anti-seizure drugs. Sedative benzodiazepines increase beta activity (36). Clobazam, an oral 1,5-benzodiazepine, is the first-line treatment drug for DS (37).

Delta is usually considered the slowest EEG frequency band (38). Delta oscillations are frequently observed in pathologic conditions, including coma (39–41) and Lennox-Gastaut syndrome (42). Increased delta activity can be observed as an ictal (43) or postictal activity (28). However, we found a decrease in delta activity in DS that might be correlated with their age. It is known that the rate of slowing increases with age in DS, suggesting that delta activity increases with the duration of epilepsy (17). Previous findings showed that SUDEP risk is increased in correlation with the duration of epilepsy (44).

In contrast to delta oscillations which usually represent pathologic conditions, gamma waves are the fastest brain waves which mainly occur when brain is highly alert and conscious. Gamma activity represents the finely-tuned inhibitory inter-neuron network (45), specifically γ-aminobutyric acid type A (GABAA) receptor-induced inhibitory postsynaptic currents (46–49). As the functional connectivity of the brain is modulated by the inhibitory inter-neuron network, dysregulation and reduction of gamma activity are observed in pathological conditions such as Alzheimer’s disease (50). A normal gamma frequency represents cognitive functions including sensory processing (51, 52), recognition, and memory (53). Modifications of gamma oscillations have been reported in mouse models of Alzheimer’s disease (in which NaV1.1 expression is reduced, leading to epileptiform activities) (54).

This study had some limitations. As our center was a tertiary referral epilepsy center, only patients with severely drug-resistant epilepsy were included. This could have caused a selection bias. In addition, we used a SUDEP-7 inventory score in our investigation instead of the actual event because SUDEP is not a condition that can be confirmed. However, we still think this study is valuable because this seven-item weighted inventory derived from a prospective SUDEP study (22, 55) is a validated, well-known tool which has been used frequently to identify risk factors for SUDEP (55–57). Also, a broad range of age (2–31 years old) was considered. In future research, it may be beneficial to compare subpopulations within different age ranges to highlight potential differences during specific life stages. This could help use this data for early intervention strategies before clinical deterioration and seizures occur.

## 5. Conclusion

Here, we identified the quantitative EEG findings that correlate with a high SUDEP-7 score. Patients with high theta band powers, and the low relative alpha band powers warrant high-level supervision.

## Data availability statement

The datasets presented in this article are not readily available because participants and guardians have not given consent for data sharing. Requests to access the datasets should be directed to SK, seheekim@yuhs.ac.

## Ethics statement

The studies involving humans were approved by the Institutional Review Board of Severance Hospital. The studies were conducted in accordance with the local legislation and institutional requirements. The Ethics Committee/Institutional Review Board waived the requirement of written informed consent for participation from the participants or the participants’ legal guardians/next of kin because this study is retrospective nature of the study.

## Author contributions

J-YK contributed to analyzing data and wrote the paper. JS collected the data. SK wrote sections of the manuscript. LK and SK supervised the study process and manuscript writing. All authors contributed to the article and approved the submitted version.

## Funding

This study was supported by the Seokcheon grant from the Korean Pediatric Society, a faculty research grant of Yonsei University College of Medicine (6-2018-0057), and the Institute of Information & Communications Technology Planning & Evaluation (IITP) grant funded by the Korean government (MSIT) (grant number 2017-0-00432) for the development of a noninvasive integrated BCI SW platform to control home appliances and external devices through an AR/VR interface.

## Conflict of interest

The authors declare that the research was conducted in the absence of any commercial or financial relationships that could be construed as a potential conflict of interest.

## Publisher’s note

All claims expressed in this article are solely those of the authors and do not necessarily represent those of their affiliated organizations, or those of the publisher, the editors and the reviewers. Any product that may be evaluated in this article, or claim that may be made by its manufacturer, is not guaranteed or endorsed by the publisher.
